# Oral Health Practices Among Pakistani Physicians

**DOI:** 10.7759/cureus.2093

**Published:** 2018-01-21

**Authors:** Syeda H Fatima, Sajida Naseem, Sara A Awan, Haider Ghazanfar, Zainab Ali, Najeeb A Khan

**Affiliations:** 1 Department of Health Profession Education, Shifa College of Medicine, Islamabad, Pakistan; 2 Department of Community and Family Medicine, Shifa International Hospital, Islamabad, Pakistan; 3 Department of Pathology, Shifa College of Medicine, Islamabad, Pakistan; 4 Internal Medicine, Shifa College of Medicine, Islamabad, Pakistan; 5 Medicine, Shifa International Hospital, Islamabad, Pakistan; 6 Family Medicine, Alam Hospital, Pakistan

**Keywords:** oral health, physicians, modified bass technique

## Abstract

Introduction

In most healthcare models, the first interaction of a patient is with a general physician. The inspection of the oral cavity is a mandatory component of the general physical examination performed by a physician. This helps detect any oral pathology and make suitable referrals. Therefore, adequate oral health awareness is essential for physicians. Our study aimed at evaluating the oral health practices among physicians working in a private teaching setup in Islamabad, Pakistan.

Methods

A cross-sectional study involving 144 physicians teaching undergraduate medical students at Shifa College of Medicine and its affiliated hospital, Shifa International Hospital, was conducted. Participants were interviewed through a self-designed questionnaire. Later, each participant demonstrated their teeth brushing technique on a standard model of the oral cavity, which was assessed against a checklist conforming to the modified bass technique. A video clip showing the aforementioned brushing technique was shown at the end of the interview. The collected data was analyzed on IBM's statistical package for the social sciences (SPSS) version 21.

Results

Toothpaste was the top choice (97.2%) of teeth cleaning tool with 69% participants brushing their teeth two times a day and 56.9% using toothbrushes with bristles of medium texture. The use of mouthwash (32.6%) and dental floss (11.1%) was considerably low. Dental caries and teeth discoloration were seen in 46.5% and 43.8% physicians, respectively. An alarmingly low number of physicians (31.9%) claimed to have read guidelines regarding oral health. This translated into most participants (78.5%) visiting a dentist only when needed. Only 4.9% participants performed all components of the modified bass technique to clean teeth on the oral cavity model, with up to 22.9% unable to perform a single step accurately.

Conclusion

The oral health knowledge and practices of physicians were found to be suboptimal and necessitate the integration of oral health awareness in the medical curriculum. A multiprofessional approach with physicians in crucial roles is required to address the burden of dental diseases globally.

## Introduction

World Health Organization (WHO) defines oral health as "a state of being free from mouth and facial pain, oral and throat cancer, oral infection and sores, periodontal (gum) disease, tooth decay, tooth loss, and other diseases and disorders that limit an individual’s capacity in biting, chewing, smiling, speaking, and psychosocial well-being" [1]. The Fédération Dentaire Internationale (FDI) World Dental Federation defines it as "multifaceted and includes the ability to speak, smile, smell, taste, touch, chew, swallow, and convey a range of emotions through facial expressions with confidence and without pain, discomfort, and disease of the craniofacial complex" [2].

Oral health is a significant challenge in today's world as in 2010, the global economic burden of dental diseases was reported to be 442 billion dollars, out of which 298 billion dollars was incurred on direct treatment, and 144 billion dollars was the indirect cost of productivity losses due to caries, periodontitis, and tooth loss [3].

Bacterial biofilm forms supragingival and subgingival plaque, which leads to periodontal diseases and dental caries. In the past, various studies have established an association of periodontal diseases with cardiovascular, respiratory, neurological, endocrine, gastrointestinal, and gestational disorders. According to a study in Spain, the modified Bass (Mod-Bass) technique was significantly more effective in removing supragingival plaque than normal toothbrushing practices, both in the buccal and lingual sites [4]. Health professionals play a pivotal role in the prevention, control, and management of oral diseases globally. Therefore, our study aimed at assessing oral health practices among physicians.

## Materials and methods

A cross-sectional study was conducted on full-time physicians working in Shifa International Hospital and Shifa College of Medicine, components of Shifa Tameer-e-Millat University, a private institution in Islamabad, Pakistan. The study spanned from December 2016 to June 2017. A sample size of 150 was determined by keeping a confidence level of 95%, a prevalence of dental caries of 16.3% [5], and an absolute precision of 6%. A list of all the physicians working in Shifa International Hospital and Shifa College of Medicine was obtained. The inclusion criteria consisted of all full-time physicians employed at Shifa International Hospital and Shifa College of Medicine. All dentists working at Shifa International Hospital and all full-time physicians on more than six months' leave were excluded. Following this, 150 physicians were selected from a computer-generated table of random numbers. After taking verbal, informed consent from the participants, the data were collected in two stages. In the first section of the self-designed questionnaire, data were collected on 23 variables. Later, participants were asked to demonstrate their tooth brushing technique on a model. A standard checklist conforming to the modified Bass technique was used to evaluate the participants. In the modified Bass technique, a toothbrush is placed such that the bristles are directed towards the base of the tooth at the gum line, with an angle of 45° to the long axis of the tooth, keeping the brush head in contact with gingiva and teeth. These movements result in the cleaning of lingual sides when the brush filaments are rolled down on the occlusal side of teeth [4]. Following the interview, a video clip showing the modified Bass technique was shown to the participants to apprise them of the standard technique.

The data obtained was entered and analyzed using SPSS version 21. Descriptive statistics were calculated. The correlation coefficient was calculated between the duration of teeth brushing of the participant and the score on teeth cleaning. The chi-square test was used to determine the difference between the modified Bass technique use, the frequency of tooth brushing per day, and caries among the two groups of doctors. A t-test of Independent samples was used to determine the difference in the mean duration of teeth brushing among the two groups of physicians. P-value <0.05 was considered statistically significant. Ethical approval of the study was obtained from Shifa International Hospital's Institutional Review Board and Ethics Committee.

## Results

Among the 144 participants, 36 (25%) were males while 108 (75%) were females. A total of 61 physicians (42.4%) were working in basic health sciences as opposed to 83 (57.6%) working in clinical health sciences. A majority of the physicians (140 (97.2%)) claimed to use only toothpaste as their main tool to clean teeth. Colgate and Sensodyne were popular choices with 77 (53.5%) and 19 (13.2%) of the participants, respectively. Most physicians specifically looked for fluoride (63 (43.8%)) in their toothpaste while the others (29 (20.1%)) valued a minty flavor more. An overwhelming majority of the participants brushed their teeth two times a day. The mean duration of tooth brushing was 2.5±1.6 minutes. A significant number of physicians (82 (56.9%)) used a medium brush type followed by the usage of the soft brush by 47 participants (32.6%). Most of the participants (51 (35.4%)) changed their toothbrush every three months, followed by 38 (26.4%) changing it after two months and only 28 (19.4%) changing it every month.

Only 47 physicians (32.6%) claimed to use mouthwash regularly, with even fewer (16 (11.1%)) using dental floss on a regular basis. About 31 (21.5%) physicians were in the habit of brushing their teeth after the consumption of sweet food. Among the participants, only 16 (11.1%) complained of teeth sensitivity to sugar as opposed to 39 physicians (27.1%) who suggested their teeth were sensitive to hot and cold temperatures. A large number of physicians had teeth discoloration (63 (43.8%)) and dental caries (67 (46.5%)).

Only 14 (9.7%) participants were smokers in addition to 10 participants (6.9%) who consumed sheesha. None of the participants consumed paan or alcohol regularly. However, soft drinks were a relatively popular choice among physicians, with the majority consuming at least two drinks (22 (15.3%)) or one drink (21 (14.6%)0 in a week.

**Figure 1:**
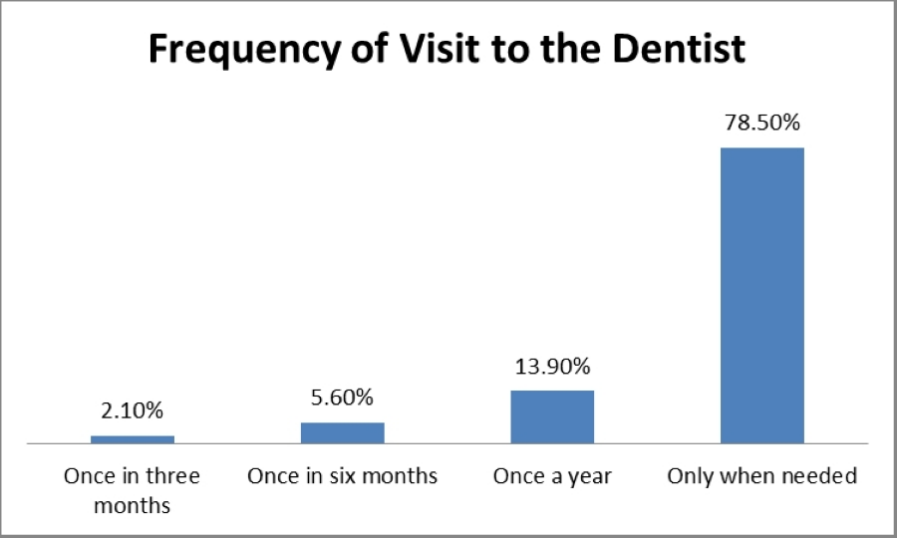
Frequency of visits to the dentist

Only an alarming 46 (31.9%) physicians claimed to have read guidelines regarding oral health. It is also of extreme concern to note that 113 physicians (78.5%) only visited a dentist when needed, 20 (13.9%) visited the dentist annually, 8 (5.6%) visited the dentist every six months, and only 3 (2.1%) visited the dentist every three months. This is represented in Figure 1.

**Figure 2:**
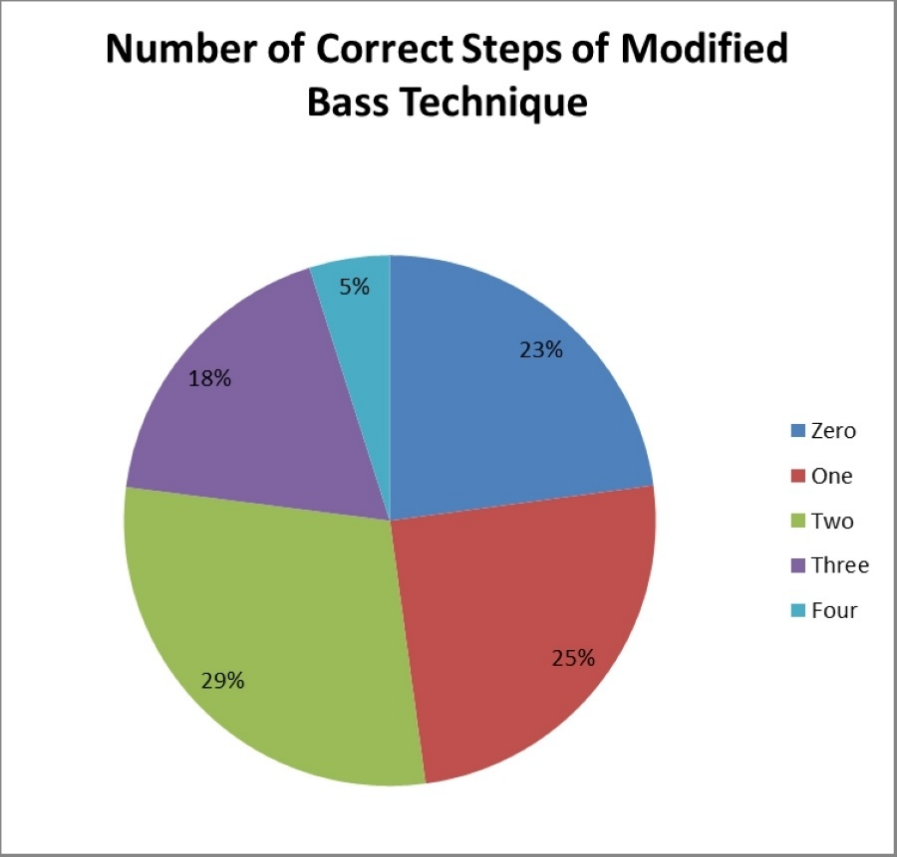
Number of correct steps of the Modified Bass technique

About 48 physicians (33.3%) held their toothbrush at a 45-degree angle while brushing teeth. While a majority (87 (60.4%)) performed brush movements in a circular motion, only 58 (40.3%) made sure the movements started from inside and moved outside. Fewer still (33 (22.9%)) slid down their toothbrush after every circular movement. Only seen participants (4.9%) performed all the four steps. Most of the participants (42 (29.2%)) performed two steps correctly while 33 (22.9%) were unable to get a single step right. This is presented in Figure 2.

When the correlation coefficient was calculated between the duration of teeth brushing of the participant and the score on teeth cleaning, no correlation was seen; p > 0.05. The chi-square test was used to determine the difference between the BASS technique and the use and frequency of tooth brushing per day with caries among the faculty of basic health sciences and clinical health sciences; the results were not statistically significant, p > 0.05. A t-test of independent samples was used to determine the difference in the mean duration of teeth brushing among the two groups of physicians; it was not statistically significant.

## Discussion

Recent years have seen an increase in emergency department (ED) visits owing to dental emergencies in America. In the year 2010 alone, Americans have visited EDs 2.1 million times with dental complaints, amounting to roughly $867 million to $2.1 billion [6]. The severity of dental diseases cannot be underestimated, as between 2000 and 2008, a total of 61,439 hospitalizations due to periapical abscesses took place in the United States, which cost the US healthcare system $858.9 million [7].

In our study participants, a significant majority (97.2%) only used toothpaste to clean teeth. This is comparable to the results obtained in a study among healthcare professionals working at King Fahad Medical Centre, Riyadh [8], where 99.4% health professionals claimed to use toothpaste only. In two other studies conducted on pharmacists in Saudi Arabia [9] and Nigeria [10], 92.5% and 100% of the participants, respectively, used toothpaste as their major teeth-cleaning tool.

Our study showed an alarmingly low use of mouthwash (32.6%) and dental floss (11.1%) in physicians. However, the results are in harmony with less than 50% health professionals using mouthwash and 34.2% using dental floss in a study in Riyadh [8]. In Nigerian doctors, the use of dental floss is closely similar (16.5%) [10]. In another study conducted on self-reported oral health practices in adults in Kuwait [11], only 11.8% claimed to use dental floss.

Our study demonstrated 69% participants brushing their teeth two times daily while 20% only brushed once a day. The average brushing time for participants was 2.5 ± 1.6 minutes. In Riyadh [8], most health professionals (77.9%) brushed their teeth once in the morning with a significant majority (50.6%) brushing for more than two minutes. In contrast, only 43.2% doctors in Nigeria [10] observed the practice of brushing teeth two times daily. This is similar to 45% pharmacists in Saudi Arabia [9] and 62% Kuwaiti adults [11] who reported brushing their teeth two times daily.

While in our study 43.8% physicians valued the presence of fluoride in their toothpaste, only 1.2% health professionals in a study in Riyadh [8] showed this preference for fluoride, which reflects their lack of awareness of the role of fluoride in dental caries prophylaxis.

The key indicators that can be used to monitor oral health include dental caries and tooth loss. According to the National Institute of Dental and Craniofacial Research, USA, 91% of US adults between the age of 20 and 64 have had dental caries in their permanent teeth in 2011-2012. In contrast to this, our study showed the prevalence of dental caries among physicians to be only 46.5%. A study carried out on Chinese urban adults aged 20-69 years demonstrated that 40.7% of the subjects reported teeth sensitivity [12]. The results are significantly lower in our research, where only 27.1% participants reported sensitivity to temperature extremes and even less (11.1%) complained of sensitivity to sweet food. These visibly better statistics in physicians as compared to the general population can be attributed to better teeth brushing techniques.

It was concluded that an overwhelming majority (78.5%) of participants in our study visited the dentist only when needed and a very small percentage (13.9%) sought annual dental checkups. This is comparable to a study on clinical medical, dental, and paramedical students in Mangalore where the result showed that 75% medical students, 86% paramedical students, and 69% dental students only visited the dentist when they encountered a dental problem (P<0.001) [13].

Regular teeth cleaning is imperative to prevent periodontal diseases and dental caries. In 2014, a study was conducted to analyze the different toothbrushing techniques recommended by dental associations, dental textbooks, and toothpaste and toothbrush companies. The results demonstrated a wide difference in opinion but, overall, the most commonly recommended technique was found to be the modified Bass technique followed by the Bass technique, Fones technique, and Scrub technique [14]. 

According to a study in Spain, the modified Bass (Mod-Bass) technique removed supragingival plaque more effectively than normal tooth brushing techniques. After 21 days, the normal practices did not significantly decrease the mean plaque indices compared to the scores calculated after seven days (P > 0.05) but did so with the modified Bass technique (P < 0.05). In the lingual side, the modified Bass technique was 2.9 times more effective than normal practices in reducing plaque scores (P < 0.01) [4]. Another study in Gulbarga on children showed that the modified Bass technique was the most effective brushing technique in removing dental plaque, followed by the horizontal scrub technique and the least effective was the Fones technique [15]. Only seven participants in our study performed the correct modified Bass technique. However, this demonstrated no significant association with the decrease in dental caries in those participants.

In a study conducted on internal medicine trainees in an urban teaching hospital located in New York City, results demonstrated that 90% trainees did not receive any training about periodontal disease in medical school, 69% could not perform a standard periodontal examination comfortably, and 76% admitted to having never screened their patients for periodontal diseases. However, it was alleviating to know that 17% physicians realized that patients expect their physician to screen them for periodontal disease and a further 46% felt a periodontal examination of the patients was imperative for the physician [16]. Similar statistics were revealed in a study on primary-care physicians in Tehran city where most respondents (95%) reported the need for a primary-care physician to know about oral health care, but 78% confessed that there is a dearth of knowledge regarding this field in physicians [17]. 

Even though there is not enough evidence on a general physician's oral health knowledge and practices in Pakistan, one study among medical doctors in Khyber Teaching Hospital and Hayatabad Medical Complex, Peshawar, showed an alarmingly low level of oral health care awareness among the participating physicians. This is comparable to the results of our study where only 31.9% doctors reported to have read guidelines regarding oral health.

## Conclusions

The oral health knowledge and practices of physicians in Pakistan is suboptimal and necessitates the integration of oral health awareness in the medical curriculum. A multiprofessional approach with physicians in crucial roles is required to address the burden of dental diseases globally.
